# Large-Scale Brain Networks Underlying Successful and Unsuccessful Encoding, Maintenance, and Retrieval of Everyday Scenes in Visuospatial Working Memory

**DOI:** 10.3389/fpsyg.2019.00233

**Published:** 2019-02-12

**Authors:** Valerio Santangelo, Cecile Bordier

**Affiliations:** ^1^Department of Philosophy, Social Sciences and Education, University of Perugia, Perugia, Italy; ^2^Neuroimaging Laboratory, Santa Lucia Foundation, Rome, Italy; ^3^Center for Neuroscience and Cognitive Systems, Istituto Italiano di Tecnologia, Rovereto, Italy

**Keywords:** working memory, everyday life scenes, frontoparietal, default mode, salience, network, Independent Component Analysis (ICA)

## Abstract

Recent research on working memory (WM) identified the contribution of several large-scale brain networks operating during WM tasks, such as the frontoparietal attention network (AN), the default mode network (DMN), and the salience network (SN). To date, however, the dynamical interplay among these networks is largely unexplored during successful or unsuccessful WM performance, especially with complex and ecological stimuli. Here we systematically characterized the selective contribution of these networks during a visuospatial WM task requiring the encoding, maintenance and retrieval of real-life scenes. While undergoing fMRI scans, participants were presented with everyday life visual scenes for 4 s (encoding phase). After a delay of 8 s (maintenance phase), participants were presented with a target-object extracted from the previous scene. Participants had to judge whether the target-object was presented at the same or in a different location compared to the original scene (retrieval phase) and then provide a confidence judgment. Using the independent component analysis (ICA), we found that subsequent remembering was associated with the activity of the AN at encoding, the attention and SN at maintenance, plus the visual network at retrieval. Conversely, subsequent forgetting was associated with the activity of the DMN at maintenance, and the SN at retrieval. Overall, these findings reveal a dynamical interplay between large-scale brain networks during visuospatial WM performance related to complex, real-life stimuli.

## Introduction

Research on working memory (WM) processes has recently benefit from the chance to use a multivariate - data-driven - approach to investigate whole brain activity co-variation (e.g., Golland et al., 2008; Cohen and D’Esposito, 2016; see, for reviews, Calhoun et al., 2009; Raichle, 2016). This approach allowed identifying the contribution of several large-scale brain networks that operate during WM tasks, such as the frontoparietal attention network (also called Attention network, AN; Duncan, 2013), the default mode network (DMN; Raichle, 2015), and the salience network (SN; Uddin, 2015). However, these networks were typically identified by means of the classical n-back task (see, for a meta-analysis, Owen et al., 2005), which is a “continuous” WM task that involves an overlap between the different WM phases, namely, encoding, maintenance and retrieval. As such, the n-back task did not allow disentangling the contribution of large-scale brain networks during each WM phase. Recently, this issue has partially been addressed by Piccoli et al. (2015). They used a delayed visuo-spatial WM task to assess the interplay between two specific large-scale brain networks, the AN and the DMN, across the three memory phases. In this study, participants were presented at encoding with a sample memory set consisting of one, three, or five colored circles (i.e., at different WM load conditions) for 2 s. The circles were randomly presented at 12 possible locations along a circumference and in three different colors: blue, yellow, or red. After a delay period ranging between 9 and 12 s, a white target circle was presented, randomly located in one of the 12 positions. Participants had to decide whether the target stimulus was or was not included in the previous sample. Piccoli and colleagues analyzed the functional connectivity during each WM phase between the AN and DMN, showing that the two networks were negatively correlated during the maintenance phase, but not during the encoding and the retrieval phase. Conversely, the activity of the AN and the DMN was positively correlated.

While providing important insights into the dynamical relationship of the AN and DMN according to WM phase, this previous literature leaves open the question of whether the increased activity in the DMN (together with the decreased activity in the AN) is explicitly associated with erroneous WM performance during the maintenance phase. This previous literature also neglected to investigate the dynamical contribution of other networks that are known to play a major role in WM processes, such as the SN (e.g., Menon and Uddin, 2010), and, subsequently, its dynamical relationship with the AN and DMN during WM performance. Previous literature demonstrated that - at rest - both the AN and the SN concurrently coordinate information processing by regulating activity within the DMN (Chen et al., 2013). However, how the dynamical interplay between these networks is related to the different WM phases and performance outcomes (i.e., successful vs. unsuccessful) is entirely unexplored to date. Finally, the previous literature typically investigated large-scale brain networks supporting WM processes using very simple and repetitive stimuli (such as geometrical shapes; e.g., Piccoli et al., 2015), while it would be critical nowadays to investigate cognitive processes using more realistic stimuli, thus to mimic the involvement of cognition in complex and real-life situations (e.g., Felsen and Dan, 2005; Peelen and Kastner, 2014).

Here we used a delayed match-to-sample WM task based on everyday life scenes. Our main aim was to systematically characterize the contribution of large-scale brain networks during successful or unsuccessful encoding, maintenance or retrieval of everyday scenes. Following a common approach in memory research (see, for a review, Kim, 2011), we distinguished WM trials according to participants’ performance, thus to investigate large-scale brain networks supporting either subsequent remembering or subsequent forgetting during each WM phase. At encoding, participants were presented with everyday life scenes. After a delay period, participants were presented with a target object extracted from the previous scene. The target object could be either highly “salient” from a perceptual point of view (in terms of local discontinuities in line orientation, intensity contrast, and color opponency; Itti and Koch, 2001) or not. Participants had to judge whether the target object was presented at the same or in a different location as compared to the original scene (i.e., a visuo-spatial WM task). To truly tap into WM retrieval and to rule out any response based on a mere sense of familiarity (Epstein and Kanwisher, 1998; Davachi, 2006; Diana et al., 2007), we asked participants to provide a confidence judgment after the localization task, indicating whether they were sure or not about their “spatial” response. Only correct spatial localizations followed by “sure” confidence judgments were classified as remembered trials, while wrong localizations or correct localizations followed by “unsure” responses were classified as forgotten trials.

We used the analysis of the independent components (ICs; see Beckmann, 2012) to highlight large-scale brain networks operating during our visuo-spatial WM task. To show the specific contribution of these networks in sustaining successful or unsuccessful encoding, maintenance and retrieval, we used a hierarchical approach: first, we assessed which ICs were recruited during our task, irrespective of WM phase (encoding, maintenance or retrieval) and WM performance (remembered or forgotten trials). Next we looked for networks sensitive to memory phase by testing the fitting of the IC time course on “encoding > maintenance and encoding > retrieval,” “maintenance > encoding and maintenance > retrieval,” and “retrieval > encoding and retrieval > maintenance”; finally, we tested the fitting of phase-specific ICs on “remembered > forgotten events” (subsequent remembering) or on “forgotten > remembered events” (subsequent forgetting), and whether phase-specific ICs were influenced by the type of the to-be-remember stimulus, salient vs. not salient. Based on the existent literature, we would expect that - irrespective of the stimulus type - the AN is positively associated with successful WM performance, across the three WM phases. A similar prediction might be extended to the SN that is thought to coordinate - along with the AN - other neural resources for processing stimuli that might be potentially relevant for goal-directed behavior (Menon and Uddin, 2010; Chen et al., 2013). Conversely, we would expect that the recruitment of the DMN during WM maintenance is associated with erroneous WM performance (Piccoli et al., 2015).

## Materials and Methods

### Participants

A total of 16 healthy volunteers took part in the fMRI experiment (all right-handed; seven males; mean age, 24.3 ± 3.5 years; range, 19–32 years). All participants were naïve university students, who gave informed and written consent to the study, which was approved by the independent Ethics Committee of the Santa Lucia Foundation. Participants had no history of head injury or physical, neurological, or psychiatric illness. The experiment was conducted in accordance with the ethical standards of the 1964 Declaration of Helsinki (last update: Seoul, 2008).

### Stimuli and Task

**FIGURE 1:**
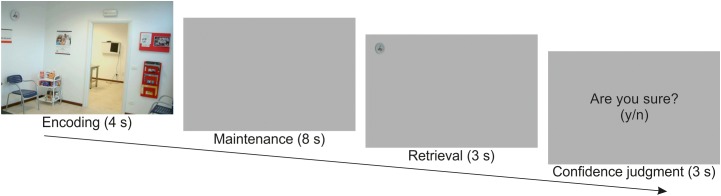
Schematic diagram illustrating the sequence of events in one example trial. Participants were presented with a scene for 4 s. After a maintenance interval of 8 s, they were presented with a target object extracted from the previous scene. They had to judge whether the target object was placed in the same or in a different location with respect to the original scene (3 s), and then provide a confidence judgment about the previous response (3 s).

Stimuli and task were fully described in Santangelo and Macaluso (2013), wherein we reported standard univariate analyses of this data set. Briefly, the WM task consisted in an encoding phase (4 s), a maintenance phase (8 s), a retrieval phase (3 s), and, finally, in a memory confidence judgment (3 s; Figure 1). At encoding, subjects were presented with pictures depicting scenes of everyday life. The picture set consisted in one hundred images collected on the World Wide Web, including either internal (e.g., a kitchen, a bathroom, etc.) or external scenarios (e.g., a garden, a street, etc.), but no single-object photo or living things such as people or animals. Each picture was analyzed with the Saliency Toolbox 2.2^1^ that computed saliency maps using local discontinuities in line orientation, intensity contrast, and color opponency (Itti et al., 1998). For half of the pictures, we designated as “target object,” the object located at the point of maximal salience (i.e., high-saliency targets), while for the other half we designated the object located at the point of minimal salience (i.e., low-saliency targets).

Participants were required to memorize as many details as possible for later retrieval. The identity of the target object was unknown until retrieval (i.e., a delayed match-to-sample task). Following a 8 s delay consisting of a blank screen, participants were shown a single target object cut out from the original picture and presented on a gray background (retrieval phase). In half of the trials, the target object was presented at the same location as in the original image, while in the other half of the trials the target object was presented at the mirror location in the opposite hemifield. Participants had to report whether the target object was in the “same” versus “different” location with respect to the position at encoding by pressing one of two response buttons. Participants were then required to express a confidence judgment concerning their response. For this, a display with the question “Are you sure? (y/n)” was presented, and participants pressed again one of the two response buttons. This was followed by a variable intertrial interval (1–3 s, uniformly distributed). Participants underwent two fMRI runs including 50 trials and lasting 16.5 min each.

### fMRI Methods

#### Image Acquisition

A Siemens Allegra (Siemens Medical Systems, Erlangen, Germany) operating at 3T and equipped for echo-planar imaging (EPI) was used to acquire the functional magnetic resonance images. A quadrature volume head coil was used for radio frequency transmission and reception. Head movement was minimized by mild restraint and cushioning. Thirty-two slices of functional MR images were acquired using blood oxygenation level-dependent imaging (3 × 3 mm, 2.5 mm thick, 50% distance factor, repetition time = 2.08 s, time echo = 30 ms), covering the entirety of the cortex. Eye-movements were also recorded during fMRI scanning (see Santangelo and Macaluso, 2013, for the analysis of eye-movements data).

#### Image Processing

We used SPM12 (Wellcome Department of Cognitive Neurology) implemented in MATLAB R2012b (The MathWorks Inc., Natick, MA, United States) for data preprocessing and GLM. Each participant underwent two fMRI-runs, each comprising 477 volumes. After having discarded the first four volumes of each run, all images were corrected for head movements. Slice-acquisition delays were corrected using the middle slice as reference. All images were normalized to the standard SPM12 EPI template, resampled to 2 mm isotropic voxel size, and spatially smoothed using an isotropic Gaussian kernel of 8 mm FWHM. Time series at each voxel for each participant were high-pass filtered at 220 s and pre-whitened by means of autoregressive model AR(1).

#### Independent Component Analysis

The main aim of the current study was to highlight the brain networks involved with subsequent remembering and subsequent forgetting during the encoding, maintenance and retrieval of everyday life scenes. These brain networks were identified by means of the independent component analysis (ICA) as implemented in the Group ICA of fMRI Toolbox (GIFT; Calhoun et al., 2001). This method involves performing ICA on functional data concatenated over every participant, creating a series of spatial maps and associated time courses for the group. The number of components was automatically estimated using a data driven approach, namely the “minimum description length” criteria, developed by Li et al. (2007). A total of 28 components were extracted. The infomax algorithm was repeated twenty times with randomly initialized decomposition matrices and the same convergence threshold using ICASSO approach in GIFT (Himberg et al., 2004). ICASSO allows for the estimation of small changes in the dataset as a result of changes in data stability. In fact, as a finite set of data never follows exactly the same ICA model, introducing ICASSO allows for estimating the reliability of the generated components. Back reconstruction was then used to create individual time courses and spatial maps from each participant’s functional data. All the ICs that involved the majority of activation falling outside the cerebral cortex, for instance in the spinal cord, eyes, borders of the skull, ventricles, etc., were considered as noisy components and excluded from further analyses. After careful visual inspection of the spatio-temporal characteristics of each IC, twelve components were categorized as noisy components (see Supplementary Figure S1), leaving 16 components for further analyses. These latter components were labeled according to the percentage of overlap with previously defined brain networks (see Supplementary Table S1 and Supplementary Figure S2), computed using the template provided by BrainMap IC^2^ (Fox and Lancaster, 2002; Laird et al., 2011). It is important noting that while the networks provided by BrainMap IC database refer to “resting state,” our dataset was acquired during a visuo-spatial WM task. Furthermore, our sample of subjects is relatively modest as compared to the resting state studies that defined the “standard” networks, such as those included in the BrainMap IC database. It is therefore not surprising that, despite a general overlap with previous-established brain networks, many of our unnoisy ICs include extra cortical regions (see Supplementary Table S1 for details).

#### Task-Related Component Identification

Components with time courses related to the experimental design were identified with a multiple regressions analysis using the temporal sorting feature of the GIFT toolbox. Individual WM performance was modeled with SPM12. Single subject models included two regressors for each memory phase (i.e., encoding, E; maintenance, M; and retrieval, R), namely, “remembered” trials (rem, followed by confident judgment, i.e., “yes, I’m sure”) and “forgotten trials” (for, including also missed responses or unsure judgments), resulting in six conditions: E_rem, E_for, M_rem, M_for, R_rem, R_for. Events at encoding, maintenance and retrieval were modeled as miniblocks, time locked at the onset of the pictures with duration of 4, 8, and 6 s, respectively. All predictors were convolved with the SPM12 hemodynamic response function.

We tested the significance of the component time courses by doing statistics on beta weights obtained after the temporal sorting, using the “Stats on Beta Weights” GIFT utility (see, for a similar approach, Santangelo, 2018). Specifically, this utility allowed us to assess the fitting of a given IC time course with the events modeled in the SPM design matrix. Our first step was to identify which components were involved with the entire task, irrespective of the different experimental conditions. For this, we performed an omnibus *F*-test among the six regressors (E_rem, E_for, M_rem, M_for, R_rem, R_for), ultimately revealing components that were overall sensitive to our task manipulation. It is important to note that the omnibus F statistic is not biased toward any specific activation pattern (main effects or interactions) and therefore constitutes a valid approach to functionally localize eloquent voxels in the current study (see, for a similar approach, Santangelo et al., 2010).

These latter components were further analyzed to highlight their selective involvement at encoding [(E > M) and (E > R)], maintenance [(M > E) and (M > R)])], and retrieval [(R > E) and (R > M)], irrespective of WM performance (see Table 1). These contrasts allowed us to assess the time course of which component - if any - fitted significantly better with the onsets of a specific WM phase modeled with the SPM design matrix (e.g., encoding) over and above the onsets of the other WM phases (e.g., maintenance and retrieval). Holm–Bonferroni’s correction was applied to account for the risk of increased false positives as a function of an increased number of ICs tested (Gaetano, 2013). Finally, for the components resulted significantly involved with WM encoding, maintenance, and/or retrieval, we compared “remembered” vs. “forgotten” trials by means of two-tailed paired *t*-tests (again corrected by Holm–Bonferroni’s procedure to account for the risk of false positives), thus highlighting networks involved with subsequent remembering (remembered > forgotten trials) or subsequent forgetting (forgotten > remembered trials). More specifically, these contrasts allowed us to highlight whether the time course of a given IC was significantly related to the onsets of, e.g., remembered vs. forgotten trials, which also means that the time course of the same IC was negatively correlated with the onsets of the opposite condition, e.g., forgotten vs. remembered trials. This procedure enabled us to highlight an overall amount of seven brain networks, operating during one or more memory phases and associated with memory performance (i.e., subsequent remembering or subsequent forgetting; see Table 2.

**Table 1:** Independent components - grouped by brain network - showing general (*F*-test) and specific (t-contrast) differences related to memory phase.

		Omnibus *F*-test	t-contrast		
	IC	E, M, R	(E > M) and (E > R) *t, p, e. s.*	(M > E) and (M > R) *t, p, e. s.*	(R > E) and (R > M) *t, p, e. s.*
**Attention Networks**					
Right executive network	6	*F*(5,90) = 8.0; ***p* < 0.001**	–4.1, **0.001**, 1.369	–2.1, 0.163, 0.646	6.1, **0.001**, 1.224
Dorsal attention network	10	*F*(5,90) = 22.1; ***p* < 0.001**	2.9, **0.041**, 1.008	–10.1, **0.001**, 3.423	7.2, **0.001**, 1.686
Precuneus/Post. parietal	11	*F*(5,90) = 6.1; ***p* < 0.001**	–3.0, **0.036**, 0.821	5.4, **0.001**, 1.667	–2.4, 0.096, 0.572
Left executive network	13	*F*(5,90) = 1.8; *p* = 0.115			
**Default Mode Networks**					
Posterior cingulate/precuneus (post DMN)	18	*F*(5,90) = 4.1; ***p* = 0.002**	1.2, 1.000, 0.348	–1.9, 0.195, 0.684	0.6, 1.000, 0.218
Ant. cingulate/medial prefrontal (ant. DMN)	19	*F*(5,90) = 59.4; ***p* < 0.001**	5.0, **0.001**, 1.588	11.8, **0.001**, 3.938	–16.7, **0.001**, 3.819
**Salience Networks**					
Medial temporal	12	*F*(5, 90) = 3.6; ***p* = 0.005**	–0.9, 1.000, 0.360	3.9, **0.001**, 1.134	–3.0, **0.022**, 0.455
Sup. Temporal/inferior parietal (post. Salience)	22	*F*(5,90) = 8.4; ***p* < 0.001**	–4.0, **0.001**, 1.376	–2.2, 0.143, 0.739	6.2, **0.001**, 1.221
Anterior insula/ACC (ant. Salience)	28	*F*(5,90) = 80.1; ***p* < 0.001**	–7.9, **0.001**, 2.786	–11.6, **0.001**, 3.920	19.5, **0.001**, 3.930
**Visual Networks**					
Medial	2	*F*(5,90) = 6.9; ***p* < 0.001**	2.6, 0.082, 0.667	5.9, **0.001**, 1.714	3.2, **0.012**, 0.675
Primary	4	*F*(5,90) = 104.9; ***p* < 0.001**	4.8, **0.001**, 1.489	–21.7, **0.001**, 8.353	16.9, **0.00**1, 4.263
Lateral	16	*F*(5,90) = 139.1; ***p* < 0.001**	–11.8, **0.001**, 4.039	–14.5, **0.001**, 4.609	26.3, **0.001**, 5.353
**Visuo-Cerebellar Network**	15	*F*(5,90) = 35.5; ***p* < 0.001**	–0.3, 1.000, 0.229	–11.2, **0.001**, 1.946	11.5, **0.001**, 2.437
**Sensorimotor**	14	*F*(5,90) = 7.4; ***p* < 0.001**	1.1, 1.000, 0.417	4.4**, 0.001**, 1.535	–5.5, **0.001**, 1.163
**Auditory**	26	*F*(5,90) < 1; n.s.			
**Cerebellar**	24	*F*(5,90) = 1.5; *p* = 0.195			


*Significant effects are marked with bold; t, *t*-value; *p*, Holm-Bonferroni corrected *p*-value; *e. s.*, effect size (Cohen’s *d*).*

**Table 2:** The 12 task-related independent components involved either with encoding, maintenance or retrieval (see Table 1) tested during “subsequent remembering” (remembered > forgotten trials, denoted by positive *T*-values) or “subsequent forgetting” (forgotten > remembered trials, denoted by negative *t*-values).

		Encoding	Maintenance	Retrieval
	IC	*t, p, e. s.*	*t, p, e. s.*	*t, p, e. s.*
**Attention Networks**				
Right executive network	6	1.7, 0.909, 0.420	3.5, **0.025**, 0.876	–0.8, 0.867, 0.211
Dorsal attention network	10	3.6, **0.030**, 0.906	3.7, **0.025**, 0.915	3.1, 0.070, 0.767
Precuneus/Post. parietal	11	–0.5, 1.000, 0.122	–1.5, 0.751, 0.379	1.8, 0.620, 0.455
**Default Mode Networks**				
Anterior cingulate/medial prefrontal (ant. DMN)	19	–3.2, 0.065, 0.801	–3.9, **0.017**, 0.978	1.5, 0.726, 0.384
**Salience Networks**				
Medial temporal	12	–1.5, 1.000, 0.384	–1.0, 1.000, 0.248	1.1, 0.867, 0.275
Superior Temporal/inferior parietal (post. Salience)	22	–0.9, 1.000, 0.214	2.4, 0.213, 0.597	–2.5, 0.180, 0.636
Anterior insula/ACC (ant. Salience)	28	1.7, 0.905, 0.437	3.6, **0.025**, 0.899	–3.8, **0.020**, 0.958
**Visual Networks**				
Medial	2	–1.4, 1.000, 0.354	–0.6, 1.000, 0.138	–0.2, 0.869, 0.042
Primary	4	1.3, 1.000, 0,322	0.3, 1.000, 0.077	3.4, **0.037**, 0.861
Lateral	16	0.1, 1.000, 0.024	3.7, **0.025**, 0.920	–1.7, 0.642, 0.429
**Visuo-Cerebellar Network**	15	3.2, 0.065, 0.801	1.8, 0.523, 0.458	3.5, **0.037**, 0.871
**Sensorimotor**	14	–0.7, 1.000, 0.175	0.1, 1.000, 0.036	1.5, 0.726, 0.372


*Components are grouped by brain network. Significant effects are marked with bold; *t*, *t*-value; *p*, Holm-Bonferroni corrected *p*-value; *e. s.*, effect size (Cohen’s *d*).*

Finally, we conducted a targeted analysis to assess whether the brain networks operating during successful encoding, maintenance and retrieval were affected by the perceptual salience (high vs. low) of the target object. For this, we created new single subject SPM models including now four regressors for each memory phase: remembered “high salient” targets (rem_H), forgotten “high salient” targets (for_H), remembered “low salient” targets (rem_L), and forgotten “lor salient” targets (for_L). Then we assessed the fitting of the time course of the ICs recruited by successful encoding, maintenance and retrieval by contrasting rem_H vs. rem_L targets.

#### IC Covariance

Next, we investigated the covariance among ICs involved with subsequent remembering and subsequent forgetting across the different WM phases. As a first step, we extracted, for each subject, the signal corresponding to the seven ICs of interest. We then computed pairwise Pearson correlations across individual ICs signals. Next, a group-level correlation matrix was computed by Fisher-transforming and averaging individual instances of the previous matrices.

#### Granger Causality Analyses

Finally, we investigated the effective connectivity among the SN, AN and DMN using the Granger Causality Analysis (GCA). This is based on the notion that, if a time-series ‘X’ causes a time-series ‘Y’, then knowledge of X should improve the prediction of Y more than information already included in the past of Y. GCA allows computing causality by comparing the variance of the residuals after an autoregressive (AR) application to the reference signal Y, with the same variance obtained when autoregression is evaluated by combining both the past values of the signal Y and the past values of the potentially causing signal X. CGA was already demonstrated to be a viable technique for analyzing fMRI time-series (Barnett and Seth, 2011). We therefore modeled directional causality among multiple time series using GCA, as implemented in the ‘Multivariate Granger Causality Toolbox’ (MVGC; Barnett and Seth, 2014).

## Results

### Behavioral Data

The analysis of accuracy revealed that participants performed well above chance (*t* = 41.5, *p* < 0.001) with an overall retrieval accuracy (“remembered” trials followed by “sure” responses) of 77.4% (see Santangelo and Macaluso, 2013, for extended behavioral results).

### Task-Related Independent Components

**FIGURE 2:**
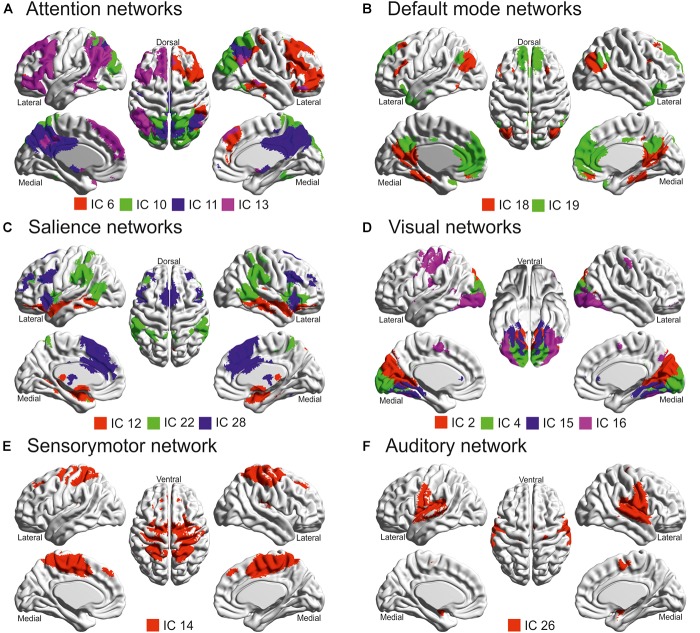
Task-related independent components (ICs) projected according to their functional similarity: **(A)** Attention networks; **(B)** Default mode networks; **(C)** Salience networks; **(D)** Visual networks; **(E)** Sensorymotor network; **(F)** Auditory network. These brain projections were created thanks to BrainNet (Xia et al., 2013). Note that the cerebellar network (IC 24) was not displayed since the cerebellum was not included in the ICBM 152 brain mesh of BrainNet (see also Table 1).

**FIGURE 3:**
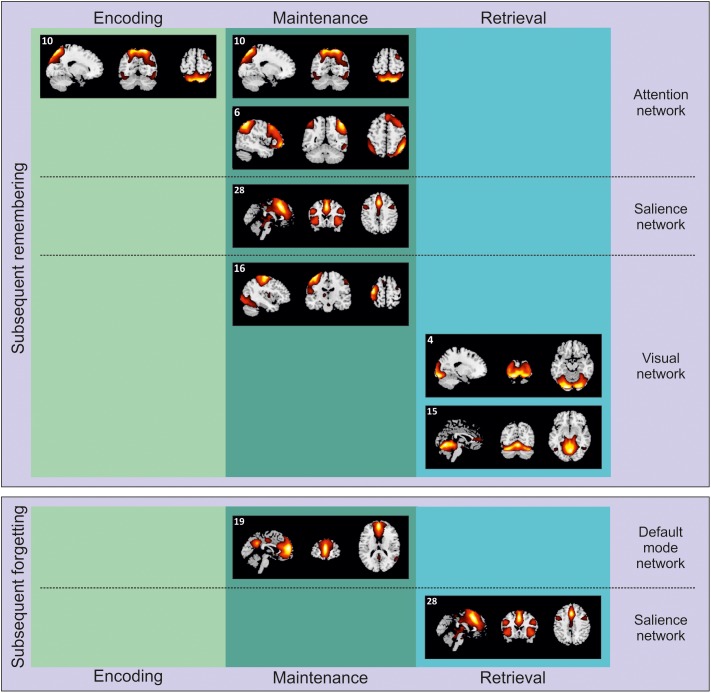
Independent components selectively involved with encoding, maintenance, and retrieval during either subsequent remembering of subsequent forgetting. The components are projected on sagittal, coronal and axial sections of a standard MNI template.

Twenty-eight ICs were identified. Of these, sixteen were determined to be task-related (i.e., not representing noise; see Figure 2 and Supplementary Table S1). Based on their main overlap with predefined networks from the BrainMap IC database (see Supplementary Figure S2), each retained component was attributed to a particular network (Damoiseaux et al., 2006; Shirer et al., 2012). These components were further analyzed in order to highlight which of them was involved with successful (remembered > forgotten) or unsuccessful (forgotten > remembered) WM performance, separately for each memory phase, encoding, maintenance and retrieval. Significant components are reported in Figure 3 and Table 1, and further analyzed below.

#### Independent Components Related to WM Encoding

The presentation of natural scenes in our task recruited several brain networks (see Table 1), including, IC 6, 10, and 11 (ANs), IC 19 (DMN), IC 22 and 28 (SN), and IC 4 and 16 (visual networks). However, the specific memory contrast (remembered vs. forgotten) drastically reduced the number of ICs involved.

##### Subsequent remembering

Successful encoding of subsequently remembered scenes was associated with the activity of IC 10 (Figure 3, top-left panel), mainly overlapping with the dorsal AN (Duncan, 2013). Posteriorly, this IC involved an extensive engagement of the posterior parietal cortex, including both superior and inferior parietal lobule, and extending to the angular gyrus, bilaterally, plus some portions of the visual cortex, such as the fusiform gyrus and the middle occipital gyrus, bilaterally. Anteriorly, IC 10 recruited the middle frontal gyrus, bilaterally. The recruitment of this IC, associated with the dorsal AN, is in line with the previous literature on WM (Piccoli et al., 2015; see also Nenert et al., 2014, for similar results on episodic memory encoding). Here we extended these findings using complex and unrepeated stimuli, i.e., everyday life scenes. Interestingly, the activity of the posterior portion of the network might be accounted for by the spatial nature of the current WM task. Successful encoding of the same pictures was already shown to recruit the posterior parietal cortex using a standard - hypothesis driven - approach (Santangelo and Macaluso, 2013). Conversely, the current data-driven approach allowed us to highlight a wider network of regions along the dorsal AN. These regions might reflect the need to encode the spatial location of objects (i.e., the possible targets at the following retrieval test) embedded in the scenes.

Despite the visual stimulation at encoding, it might be worth noting here that the analyses failed to reveal any specific contribution of the visual network. This is likely to be a consequence of a similar recruitment of the visual network irrespective of the subsequent WM performance, thus preventing this circuit to emerge in one or the other contrast (remembered vs. forgotten trials or vice-versa).

##### Subsequent forgetting

Our analysis did not reveal at the encoding any IC related to subsequent forgetting. This might suggest that the absence of a selective recruitment of the AN during WM encoding (see the previous section) could be a sufficient condition to determine a failure in WM performance.

#### Independent Components Related to Maintenance

As for the encoding phase, the maintenance phase revealed the contribution of several brain networks when tested irrespectively of memory performance (see Table 1). This included IC 10 and 11 (both part of the dorsal AN), IC 19 (anterior DMN), IC 12 and 28 (medial temporal and anterior part of the SNs), IC 2, 4, and 16 (medial, primary and lateral visual networks), IC 15 (visuo-cerebellar network), and IC 14 (sensorimotor network). Except for the sensorimotor network, the other networks were also involved with specific memory-related effects.

##### Subsequent remembering

At maintenance, several ICs were involved with successful performance (Figure 3, top-center panel). First of all, we observed again a significant contribution of IC 10, dorsal AN - already operating during successful encoding - and IC 6 (right executive network). Posteriorly, this latter network included more lateral portions (as compared to IC 10) of the superior and inferior parietal lobules, bilaterally, extending on the right hemisphere to the supramarginal and the angular gyri. Anteriorly, the IC 6 recruited larger portions of the frontal lobe than the IC 10, namely the superior, medial and inferior frontal gyri, bilaterally. Components 6 and 10 can be therefore considered as different ICs constituting the frontoparietal AN (Corbetta and Shulman, 2002; Duncan, 2013). This network was already found to contribute to successful WM maintenance (Jerde et al., 2012; Piccoli et al., 2015), aiding participants to stay focused on those elements of the visual scenes that might be target-objects at the following retrieval test.

Successful WM maintenance was also supported by IC 16, lateral visual network, that included the middle and inferior occipital gyri, extending bilaterally toward the lingual and fusiform gyri and ventrally along the adjacent inferior and middle temporal gyri. This IC also included the pre- and post-central gyri in the left hemisphere. It is worth remembering here that there was not visual stimulation at maintenance. The IC 16 might be therefore interpreted as in line with the notion that successful WM maintenance is supported by processes related to visual imagery (Keogh and Pearson, 2011; Albers et al., 2013).

Finally, our analysis highlighted a significant contribution of IC 28, mainly overlapping with the anterior SN. This network include a set of regions that are though to play a key role in selecting stimuli that are potentially relevant (Uddin, 2015). The SN is typically divided into roughly two main subdivisions: the anterior cingulate cortex and the fronto-insular cortex. Accordingly, IC 28 included the anterior cingulate cortex (extending dorsally toward the medial portions of the middle and superior frontal gyri and laterally to the adjacent prefrontal cortex) and the left and right insula. This network might further contribute - along with the AN - to WM maintenance of objects that have been selected by participants as potential targets for the upcoming retrieval test.

##### Subsequent forgetting

In line with our predictions based on the extant literature (Piccoli et al., 2015), subsequent forgetting was marked at maintenance by the recruitment of the IC 19, which involved regions known to be part of the DMN (Raichle, 2015; see Figure 3, bottom-center panel). Specifically, the IC 19 involved anteriorly the anterior cingulate cortex extending toward the ventro- and dorso-medial prefrontal cortex, and posteriorly the precuneus. Previous literature found that the DMN was negatively correlated with the AN during WM maintenance. In line with this notion, here we found that increased activity along the DMN at maintenance was associated with subsequent forgetting.

#### Independent Components Related to Retrieval

As for the maintenance phase, performance-unrelated retrieval involved IC 6 and 10 (involving different portions of the AN), IC 19 (anterior DMN), IC 12, 22, and 28 (constituting the SN), IC 2, 4, 15 and 16 (visual/visual-cerebellar networks), and IC 14 (sensorimotor network) (see Table 1). The following paragraphs highlight which of these ICs specifically contributed to memory performance.

##### Subsequent remembering

At retrieval, successful performance was supported by two components (Figure 3, top-right panel), related to the visual and visuo-cerebellar network. The IC 4 included extended portions of the occipital lobe, that is, the inferior, middle and superior occipital giri, bilaterally, plus the cuneus, the lingual and fusiform giri; the IC 15 extended more ventrally into the visual cortex, recruiting also the cerebellum. Despite the presentation of a visual stimulus (i.e., the to-be-judged target object) at retrieval, we believe that the recruitment of this ICs might be more in line with the idea that visual imagery is an important component not only at maintenance but also at retrieval (Keogh and Pearson, 2011; Albers et al., 2013). In fact, these networks were not recruited for subsequent forgetting (see next section), that is, in condition where target objects were also presented. These networks might therefore support the reconstruction of the target position within the visual scene by means of visual imagery.

##### Subsequent forgetting

Finally, our analysis revealed that unsuccessful WM performance was marked at retrieval by the enrollment of IC 28 (i.e., the anterior SN). Differently from WM maintenance, the recruitment of a sub-network of the SN at retrieval was predictive of subsequent forgetting (Figure 3, bottom-right panel). This will be further discussed in the Section “Discussion.”

#### Target Salience

The current findings revealed the recruitment of several ICs operating during successful WM performance, namely: IC 10 at encoding, IC 6, 10, 16, and 28 at maintenance, IC 4 and 15 at retrieval. We further assessed whether these networks were affected by the perceptual salience (high vs. low) of the target object. However, this analysis failed to reveal a different contribution of these networks as a function of target saliency, both at encoding (*t* = -1.289, *p* = 0.217), at maintenance (all *t*s ranging from -0.058 to 1.971, all *p*s > 0.067), and retrieval (both *t*s < 1.586, both *p*s > 0.134).

#### Independent Components Covariation

**FIGURE 4:**
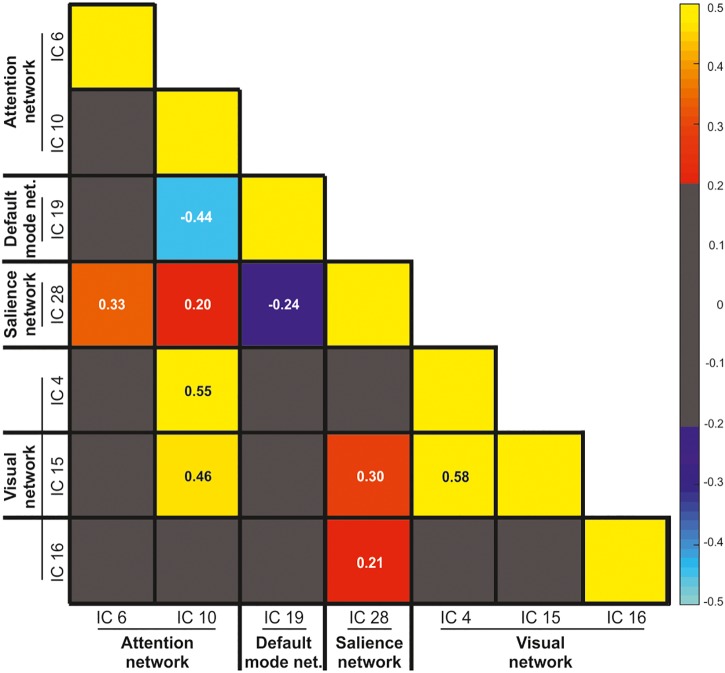
Pearson correlation values between the visual, salience, default mode, and attention networks. The gray color indicates low correlation values ranging between –0.2 and 0.2.

The ICs predicting WM performance according to the specific WM phase involved three well-known, and previously established, brain networks, namely the AN, the DMN, and the SN, plus important activity of the visual network. Figure 4 illustrates the correlation matrix among these networks, including both positive and negative correlation values. Gray cells in the correlation matrix indicates low correlation values, ranging between -0.2 and 0.2. The correlation matrix revealed a positive correlation between the IC 6 and 10 with IC 28 (e.g., AN with SN; *r* = 0.33 and *r* = 0.20, respectively). Both the AN and SN were positively correlated with the activity of the visual networks (e.g., IC 10 and 4, *r* = 0.55 and IC 28 and 4, *r* = 0.58). By contrast, and coherently with the existent literature (Chen et al., 2013), IC 19 was negatively correlated to both IC 10 (anterior DMN and dorsal AN, respectively; *r* = -0.44) and IC 28 (anterior DMN and anterior SN, respectively; *r* = -0.24), with no further correlation with the visual networks (all *r*s < 0.2).

#### Effective Connectivity Among the AN, SN and DMN

To further investigate the inter-relationship between the three main networks predicting either successful or unsuccessful WM performance across the different WM phases, namely the IC 10 (AN), the IC 19 (DMN), and the IC 28 (SN), we analyzed their effective connectivity by means of GCA. As showed in Table 3, the GCA revealed reciprocal causal relationships among the three brain networks. This means that the activity of each of these three network predicted and it was also predicted by the activity of the other two networks.

**Table 3:** Holm-Bonferroni’s corrected *p*-values derived from the Granger Causality Analysis carried out among the attention (IC 10), default mode (IC 19) and salience (IC 28) networks.

	Attention net.	—	<0.001	<0.001
To:	Default mode net.	<0.001	—	<0.001
	Salience net.	<0.001	<0.001	—
		Attention net.	Default mode net.	Salience net.
			From:	


## Discussion

The main aim of the current study was to characterize large-scale brain networks operating during successful or unsuccessful encoding, maintenance and retrieval in a visual-spatial WM task based on complex and unrepeated everyday life scenes. As expected, together with the visual network, we observed a dynamical interplay between sub-networks of the AN, the DMN and the SN across the three WM phases, depending on subsequent WM performance.

The analysis of the inter-relationship among the sub-networks of the IC constituting the AN, SN, and DMN showed reciprocal causality, indicating bidirectional influences among them. The pattern of covariation among these networks is also consistent with the previous literature. The ICs that are part of the AN and SN are positively correlated, and showed positive correlation with the visual network (Uddin, 2015). On the contrary, the activity of the IC19 (included in the anterior DMN) was negatively correlated with both the activity of the dorsal AN and the anterior SN (Chen et al., 2013; Piccoli et al., 2015) (cf. analysis of the independent components covariation). Importantly, our approach based on the estimation of the fitting of the IC time course with the GLM model allowed us to additionally assess the involvement of these brain networks across the different WM phases and performance. The recruitment of part of the AN (i.e., the IC 10) during both the encoding and the maintenance phase was associated with successful WM performance. The involvement of the areas of the AN during successful visual-spatial WM encoding and maintenance is in good agreement with the previous literature (Piccoli et al., 2015). However, here we showed that together with the recruitment of the AN, other brain networks are necessary for successful WM performance, such as the SN and the visual cortex at maintenance.

The SN has key nodes in the insular cortex and is thought to be critical for detecting stimuli that are potentially relevant from a behavioral point of view (Menon and Uddin, 2010). The current involvement of the SN at maintenance might therefore reflect the complexity of the stimuli used in the present study. Everyday life scenes include a number of objects that could be presented at retrieval as possible targets for the same vs. different location task. Accordingly, only a limited number of these objects can be encoded and maintained by participants, due to intrinsic limitations of WM (Luck and Vogel, 2013). Across several previous studies (Santangelo and Macaluso, 2013; Pedale and Santangelo, 2015; Santangelo et al., 2015) we demonstrated that the probability to retrieve a target-object embedded in an everyday scene is a function of the object-related sensory salience, that is, of low-level sensory features characterizing the specific target-object tested at retrieval. When the number of potential targets overcomes WM capacity, e.g., during the encoding of everyday life scenes, highly salient objects have more chances than lower salience objects to be successfully encoded and then correctly remembered at retrieval (see, for a review, Santangelo, 2015). Highly salient objects were shown to be more attentional capturing than lower saliency objects (Itti and Koch, 2001). As a consequence, they might have a prioritized access to perceptual and post-perceptual processes (i.e., WM maintenance and retrieval). The posterior parietal cortex was found to play a key role in the prioritization of salient objects (Nardo et al., 2011, 2014; see, for a review, Gottlieb, 2007). Although we did not find a selective contribution for remembering high vs. low salience objects (cf. target saliency analysis), the general pattern of the IC analysis is in line the previous literature. In fact, our findings revealed a massive engagement of the AN during the encoding, and especially of the posterior network nodes (i.e., the posterior parietal cortex) that might have significantly contributed to the selection of possible targets according to their current perceptual salience. These posterior nodes were still active at maintenance, along with the anterior AN nodes (i.e., the prefrontal cortex) and the SN. Together, the anterior AN and the SN might not only play a crucial role in maintaining the selected objects potentially relevant for the following memory test, but also in coordinating neural resources for the following behavioral response at retrieval (see, e.g., Lamichhane and Dhamala, 2015; Chand and Dhamala, 2016). Activity along the anterior AN and SN might be necessary for the recruitment of the visual network, to promote visual imagery/visual representation of the encoded scene to select the correct response.

Moreover, these networks might play a key role in reducing the involvement of the DMN during WM maintenance. Previous literature showed a causal relationship between increased activation of the AN/SN and decreased activation of the DMN (Chen et al., 2013). This is also compatible with the findings of Piccoli et al. (2015) reporting negative correlation between the AN and DMN during WM maintenance. Consistently with this literature, the current data showed mutual causal relationships among the AN, SN and DMN, and negative correlations between the AN/SN and the DMN. In particular, our findings appears to emphasize the role of SN, which might also contribute - together with the AN - to down regulate the DMN. This was supported by the evidence that we found an enrollment of the DMN only when the AN and SN were not involved, that is, during the maintenance phase of subsequent forgotten trials. The DMN was showed to be recruited by “internal” cognitive processes, such as mind wandering (Mason et al., 2007), self-awareness streaming (Vanhaudenhuyse et al., 2010), and autobiographic memory (Svoboda et al., 2006). As such, the DMN might be entirely unnecessary (and even detrimental) at maintenance, wherein participants are trying to preserve the representation of the external information relevant to correctly perform the visual-spatial task. The down regulation operated by the AN/SN on the DMN at retrieval might be therefore functional to keep maintenance operations undisturbed. Accordingly, we found that the enlistment of the DMN at maintenance was associated with unsuccessful WM performance, consistently with the previous literature (Piccoli et al., 2015).

Finally, our findings revealed for the first time - at last in our knowledge - that the SN might have a different impact on WM performance according to the specific WM phase. In fact, while the engagement of the SN at maintenance is associated with subsequent remembering, the recruitment of the SN at retrieval is related to subsequent forgetting. We speculate that this late enrollment of the SN might be due to an inefficient attempt to activate the network that coordinates internal stimulus representation (i.e., the AN, which is not recruited in subsequent forgotten trials) through the visual network.

## Current Limitations

An intrinsic problem related to the adoption of paradigms based on the delayed-match-to-sample task is that it is not possible to estimate *a priori* the amount of remembered and forgotten trials. This typically results in an unbalanced amount of trials used to estimate the BOLD signal in the two conditions, just as in the current experiment, with an average of 77 remembered and 23 forgotten out of 100 total trials. Future research should develop new strategies to overcome this potential flaw.

Another potential limitation is related to the fact that the current study cannot account for the possible impact of sex differences on the neural mechanisms highlighted here. Previous literature suggests that there may be crucial differences between males and females in visuospatial WM (see, e.g., Loring-Meier and Halpern, 1999; Palmiero et al., 2016). Future research involving larger samples of participants might address this interesting issue.

It is also worth mentioning here that a different approach for the ICA would have consisted in averaging signals across voxel applying masks corresponding to well-established brain networks, such as, e.g., the BrainMap IC database (Fox and Lancaster, 2002; Laird et al., 2011). While this approach has the advantage to allow a more direct comparison with previously established brain networks, it has the disadvantage to consider signals not necessarily related to the group of subjects under investigation. Here we have chosen to investigate specific ICs originating from our group of participants, despite these networks only partially overlapped with the brain networks highlighted by the previous literature.

## Conclusion

This study characterized the dynamical interplay between the large-scale brain networks operating during a visuospatial WM task based on everyday life scenes. We found that the recruitment of the AN at encoding, the AN and the SN at maintenance, plus the visual network at retrieval, are associated with subsequent remembering. Conversely, subsequent forgetting was associated with the engagement of the DMN at maintenance, and the SN at retrieval. Altogether, these findings highlight the importance to investigate the dynamical interplay between large-scale brain networks sustaining complex cognitive processes, such as WM encoding, maintenance and retrieval of real-life scenes.

## Author Contributions

VS conceived the study and collected the data. VS and CB analyzed the data and wrote the manuscript.

## Conflict of Interest Statement

The authors declare that the research was conducted in the absence of any commercial or financial relationships that could be construed as a potential conflict of interest.

## Footnotes

1 http://www.saliencytoolbox.net/

2 http://www.brainmap.org/icns/fig2.html
