# Supplementary material for: Large-Scale Brain Networks Underlying Successful and Unsuccessful Encoding, Maintenance, and Retrieval of Everyday Scenes in Visuospatial Working Memory
Source: Front Psychol. 2019 Feb 12;10:233. doi: 10.3389/fpsyg.2019.00233 (PMC6379313; doi:10.3389/fpsyg.2019.00233)
Supplement: Supplementary file 1 [file Table_1.pdf]

Large-scale brain networks underlying successful and unsuccessful encoding, maintenance, and retrieval of everyday scenes in visuospatial working memory

Valerio Santangelo[1,2] and Cecile Bordier[3]

1) Dept. of Philosophy, Social Sciences and Education, University of Perugia, Italy
2) Neuroimaging Laboratory, Santa Lucia Foundation, Rome, Italy
3) Center for Neuroscience and Cognitive Systems, Istituto Italiano di Tecnologia, Rovereto, Italy

SUPPLEMENTARY MATERIAL

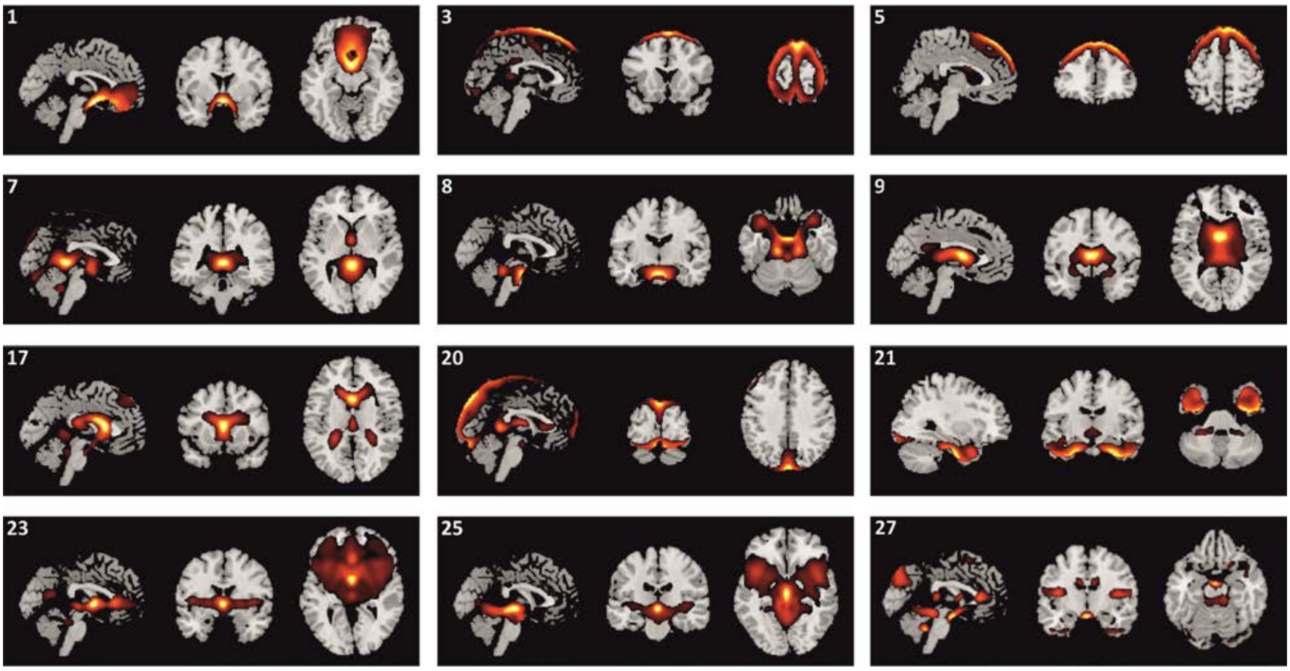

Figure S1: Discarded independent components.

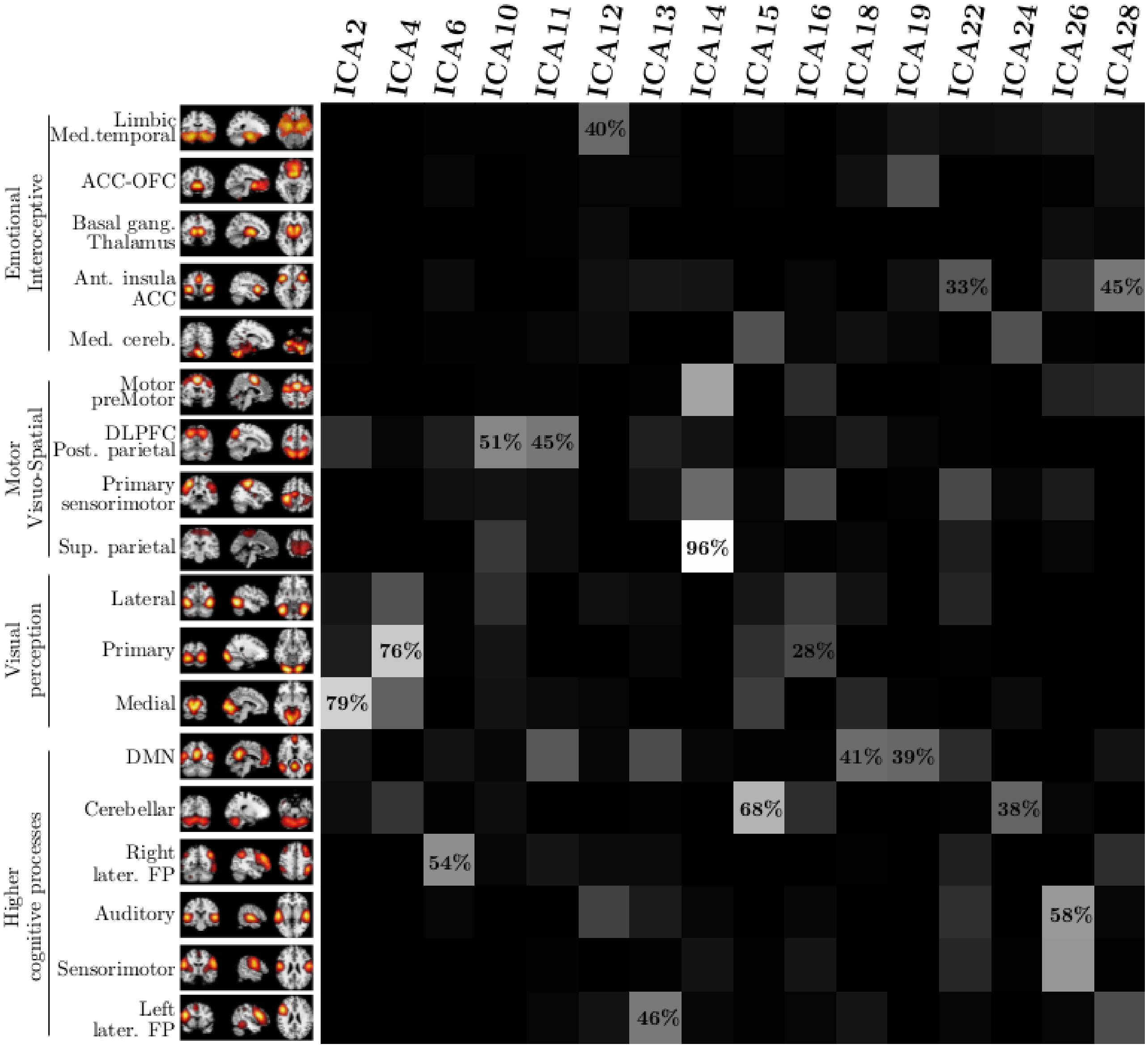

Figure S2: Percentage of overlap of the 16 unnoisy independent components (IC) with the correspondent BrainMap IC.

Table S1: Anatomical localizations, corresponding Brodmann area(s), volume (cc), maximum Z-scores and Talairach coordinates (obtained using the Talairach utility provided in GIFT toolbox on group-ICA components maps) of task-related independents components (IC).

Brain network	*IC*	*Area*	*Brodmann Area*	*Volume (cc)*	*Max Z-score & Talairach (x, y, z) L/R*
Attention	6	Inferior Parietal Lobule	7, 40	NA/3.7	NA/5.5 (48, -50, 52)
		Middle Frontal Gyrus	8, 9, 10, 11	NA/2.6	NA/4.6 (46, 48, -11)
		Superior Parietal Lobule	7	NA/0.4	NA/4.6 (42, -58, 51)
		Superior Frontal Gyrus	10	NA/0.4	NA/4.1 (36, 60, -5)
		Inferior Frontal Gyrus	10	NA/0.1	NA/3.7 (50, 44, -11)
		Supramarginal Gyrus	*	NA/0.1	NA/3.6 (51, -51, 36)
	10	Precuneus	7, 19	0.7/1.3	4.4 (-6, -61, 62)/5.3 (16, -63, 62)
		Superior Parietal Lobule	7	1.1/1.9	4.5 (-18, -67, 57)/5.0 (20, -69, 57)
		Postcentral Gyrus	7	NA/0.1	NA/4.0 (10, -53, 67)
		Cuneus	19	NA/0.1	NA/3.6 (24, -86, 37)
	11	Precuneus	7, 31	7.9/8.1	7.5 (0, -64, 46)/7.3 (4, -70, 42)
		Cingulate Gyrus	23, 24, 31	4.4/4.7	6.3 (0, -35, 29)/6.6 (2, -39, 30)
		Cuneus	7	0.4/0.3	5.4 (-4, -66, 33)/4.1 (4, -76, 37)
		Posterior Cingulate	23, 30	0.3/0.4	4.8 (-2, -43, 24)/4.9 (2, -42, 22)
		Superior Parietal Lobule	7	NA/0.1	NA/3.9 (8, -67, 53)
		Inferior Parietal Lobule	40	NA/0.1	NA/3.6 (42, -62, 45)
	13	Inferior Parietal Lobule	7, 39, 40	2.1/NA	4.5 (-50, -58, 38)/NA
		Angular Gyrus	39	0.8/NA	4.4 (-50, -59, 34)/NA
		Supramarginal Gyrus	*	0.4/NA	4.2 (-51, -59, 31)/NA
		Middle Temporal Gyrus	39	0.3/NA	3.8 (-61, -41, -1)/NA
		Superior Temporal Gyrus	39	0.3/NA	3.8 (-51, -59, 27)/NA
		Inferior Frontal Gyrus	47	0.3/NA	3.8 (-50, 21, -8)/NA
		Superior Parietal Lobule	7	0.1/NA	3.7 (-40, -62, 51)/NA
		Middle Frontal Gyrus	*	0.1/NA	3.6 (-48, 42, -9)/NA
Default mode	18	Posterior Cingulate	23, 29, 30, 31	2.6/2.9	5.1 (0, -53, 19)/5.4 (12, -54, 16)
		Sub-Gyral	*	0.2/0.4	4.8 (-10, -57, 18)/4.7 (12, -57, 19)
		Extra-Nuclear	*	0.1/0.2	3.9 (-12, -55, 21)/4.7 (18, -55, 19)
		Precuneus	23, 31	1.1/0.5	4.4 (-4, -59, 21)/4.1 (12, -59, 23)
		Cingulate Gyrus	31	0.2/0.1	4.1 (0, -57, 27)/3.8 (4, -59, 27)
		Parahippocampal Gyrus	*	NA/0.1	NA/3.6 (10, -46, 4)
	19	Medial Frontal Gyrus	9, 10	1.9/1.3	4.6 (-4, 51, 9)/4.4 (4, 53, 10)
		Anterior Cingulate	32	0.6/0.2	4.4 (-4, 47, 9)/4.3 (4, 49, 9)
		Precuneus	31	0.1/NA	3.8 (0, -49, 32)/NA
		Cingulate Gyrus	*	0.1/NA	3.6 (0, -49, 28)/NA
Salience	12	Superior Temporal Gyrus	22, 38	4.4/3.8	8.8 (-42, 9, -17)/8.3 (40, 13, -19)
		Middle Temporal Gyrus	21, 38	1.0/2.3	6.9 (-48, 5, -15)/6.2 (48, 7, -15)
		Sub-Gyral	13, 21	1.5/1.8	6.6 (-46, -3, -13)/6.1 (46, 3, -15)
		Uncus	28, 34	0.6/0.5	5.3 (-32, 3, -19)/6.5 (28, 9, -21)
		Inferior Frontal Gyrus	47	0.6/0.6	5.1 (-34, 13, -16)/6.5 (36, 13, -17)
		Third Ventricle	*	NA/0.2	NA/6.1 (0, -6, -8)
		Parahippocampal Gyrus	34	0.4/0.3	4.4 (-30, -1, -18)/5.1 (24, 3, -17)

		Extra-Nuclear	*	0.1/NA	4.0 (-4, -6, -8)/NA
		Anterior Cingulate	*	0.1/NA	3.7 (0, 5, -10)/NA
	22	Superior Temporal Gyrus	22, 38	NA/0.4	NA/4.4 (50, 17, -9)
		Inferior Frontal Gyrus	47	NA/0.3	NA/4.0 (46, 15, -7)
		Inferior Parietal Lobule	40	0.3/NA	3.7 (-59, -35, 31)/NA
	28	Cingulate Gyrus	32	0.8/0.8	4.9 (0, 29, 32)/4.6 (4, 21, 41)
		Medial Frontal Gyrus	6, 8, 9	0.4/0.5	4.1 (-4, 22, 43)/4.5 (4, 25, 39)
		Superior Temporal Gyrus	38	0.2/0.1	4.2 (-48, 15, -9)/3.7 (48, 17, -11)
		Superior Frontal Gyrus	8	0.2/0.3	3.7 (-2, 20, 51)/4.2 (2, 20, 51)
		Anterior Cingulate	32	0.4/0.3	3.9 (0, 34, 20)/4.1 (4, 32, 24)
		Inferior Frontal Gyrus	47	0.1/NA	3.9 (-44, 15, -9)/NA
Visual	2	Cuneus	7, 17, 18, 19, 23, 30	8.9/7.9	7.2 (-2, -69, 9)/7.2 (2, -73, 13)
		Lingual Gyrus	18, 19	4.2/3.9	6.9 (-10, -60, 5)/5.8 (4, -72, 4)
		Posterior Cingulate	23, 30, 31	2.4/2.5	6.5 (-6, -69, 13)/6.9 (6, -69, 11)
		Precuneus	7, 19, 31	1.0/2.0	4.8 (-2, -69, 20)/6.0 (6, -69, 18)
		Culmen	*	0.6/0.3	5.5 (-8, -56, 1)/4.2 (16, -49, -3)
		Extra-Nuclear	*	NA/0.3	NA/4.6 (24, -58, 7)
		Parahippocampal Gyrus	19, 30	0.3/0.4	4.3 (-12, -48, 4)/4.5 (14, -50, 4)
	4	Fusiform Gyrus	18, 19	1.6/1.0	6.2 (-18, -90, -11)/5.7 (20, -88, -11)
		Inferior Occipital Gyrus	17, 18	2.4/2.0	6.1 (-14, -92, -9)/5.8 (24, -88, -11)
		Lingual Gyrus	17, 18	6.0/5.0	5.9 (-18, -94, -9)/5.6 (16, -88, -9)
		Cuneus	17, 18	2.6/2.0	5.3 (-12, -97, 0)/5.1 (12, -95, 1)
		Middle Occipital Gyrus	18, 19	2.5/1.0	5.1 (-22, -86, -7)/4.9 (26, -84, -8)
		Declive	*	0.4/0.1	4.1 (-34, -82, -16)/3.8 (12, -80, -11)
		Sub-Gyral	*	0.1/0.0	3.7 (-24, -91, 0)/-999.0 (0, 0, 0)
	16	Precentral Gyrus	4, 6	2.5/NA	5.7 (-40, -14, 62)/NA
		Postcentral Gyrus	1, 2, 3, 4	2.8/NA	4.8 (-42, -15, 54)/NA
		Middle Frontal Gyrus	*	0.1/NA	3.7 (-32, -7, 61)/NA
Visuo-cereb.	15	Culmen	*	0.3/0.7	3.7 (-32, -51, -19)/4.2 (4, -66, -7)
		Declive	*	0.6/0.4	3.9 (-30, -71, -18)/4.1 (42, -57, -22)
		Culmen of Vermis	*	0.3/0.1	4.0 (0, -66, -7)/4.0 (4, -62, -5)
		Tuber	*	NA/0.1	NA/3.7 (44, -54, -24)
		Declive of Vermis	*	NA/0.1	NA/3.6 (2, -74, -10)
Sensori-motor	14	Medial Frontal Gyrus	6, 32	1.9/2.7	5.8 (-2, -3, 48)/6.2 (2, -3, 48)
		Cingulate Gyrus	24, 32	1.0/1.6	5.5 (-2, 0, 46)/5.7 (2, 0, 44)
		Paracentral Lobule	5, 6, 31	0.3/0.4	4.0 (-2, -11, 45)/4.2 (2, -11, 45)
		Superior Frontal Gyrus	6	0.1/0.1	4.1 (-2, 6, 48)/4.2 (2, 6, 49)
		Postcentral Gyrus	1, 2, 3	NA/0.6	NA/3.9 (34, -32, 61)
		Precentral Gyrus	4	NA/0.3	NA/3.7 (40, -19, 56)
Auditory	26	Superior Temporal Gyrus	13, 21, 22, 41, 42	7.1/6.5	6.5 (-59, -7, 6)/7.8 (61, -9, 6)
		Transverse Temporal Gyrus	41, 42	1.6/1.2	5.8 (-59, -9, 10)/6.6 (61, -17, 10)
		Precentral Gyrus	4, 6, 13, 43	3.1/4.0	6.2 (-59, -3, 13)/6.6 (57, -11, 10)
		Insula	13, 40	1.9/2.8	4.8 (-44, -13, 6)/5.5 (46, -13, 8)
		Postcentral Gyrus	40, 43	0.6/1.2	5.2 (-57, -7, 17)/5.3 (61, -17, 14)
		Middle Temporal Gyrus	21	NA/0.1	NA/4.3 (61, -4, -3)

		Parahippocampal Gyrus	*	NA/0.1	NA/3.8 (20, -1, -17)
Cerebellar	24	Fourth Ventricle	*	NA/0.5	NA/14.3 (0, -48, -26)
		Culmen	*	1.2/1.7	8.5 (-4, -44, -20)/11.7 (4, -44, -21)
		Nodule	*	0.6/0.8	10.0 (-4, -48, -30)/11.5 (0, -48, -30)
		Fastigium	*	0.3/0.3	6.7 (-4, -52, -21)/7.1 (8, -48, -21)
		Cerebellar Lingual	*	0.1/0.3	3.9 (-4, -45, -16)/5.3 (0, -44, -16)
		Cerebellar Tonsil	*	0.1/0.1	4.5 (-24, -33, -34)/3.9 (24, -33, -32)
		Declive	*	0.1/0.3	3.6 (-4, -63, -20)/3.7 (0, -63, -22)
